# Herpes simplex virus and Cytomegalovirus reactivation among severe ARDS patients under veno-venous ECMO

**DOI:** 10.1186/s13613-019-0616-6

**Published:** 2019-12-23

**Authors:** Sami Hraiech, Eline Bonnardel, Christophe Guervilly, Cyprien Fabre, Anderson Loundou, Jean-Marie Forel, Mélanie Adda, Gabriel Parzy, Guilhem Cavaille, Benjamin Coiffard, Antoine Roch, Laurent Papazian

**Affiliations:** 1 Service de Médecine Intensive - Réanimation, APHM, Hôpital Nord, Marseille, France; 2 CEReSS-Center for Studies and Research on Health Services and Quality of Life EA3279, Aix-Marseille University, Marseille, France; 3 Magellan Medico-Surgical Center, South Department of Anaesthesia and Critical Care, CHU Bordeaux, 33000 Bordeaux, France

**Keywords:** ExtraCorporeal Membrane Oxygenation, Acute respiratory distress syndrome, Herpes simplex virus reactivation, Cytomegalovirus reactivation

## Abstract

**Background:**

*Herpesviridae* reactivation among non-immunocompromised critically ill patients is associated with impaired prognosis, especially during acute respiratory distress syndrome (ARDS). However, little is known about herpes simplex virus (HSV) and Cytomegalovirus (CMV) reactivation occurring in patients with severe ARDS under veno-venous extracorporeal membrane oxygenation (ECMO). We tried to determine the frequency of *Herpesviridae* reactivation and its impact on patients’ prognosis during ECMO for severe ARDS.

**Results:**

During a 5-year period, 123 non-immunocompromised patients with a severe ARDS requiring a veno-venous ECMO were included. Sixty-seven patients (54%) experienced HSV and/or CMV reactivation during ECMO course (20 viral co-infection, 40 HSV alone, and 7 CMV alone). HSV reactivation occurred earlier than CMV after the beginning of MV [(6–15) vs. 19 (13–29) days; *p* < 0.01] and after ECMO implementation [(2–8) vs. 14 (10–20) days; *p* < 0.01]. In univariate analysis, HSV/CMV reactivation was associated with a longer duration of mechanical ventilation [(22–52.5) vs. 17.5 (9–28) days; *p* < 0.01], a longer duration of ECMO [15 (10–22.5) vs. 9 (5–14) days; *p* < 0.01], and a prolonged ICU [29 (19.5–47.5) vs. 16 (9–30) days; *p* < 0.01] and hospital stay [44 (29–63.5) vs. 24 (11–43) days; *p* < 0.01] as compared to non-reactivated patients. However, in multivariate analysis, viral reactivation remained associated with prolonged MV only. When considered separately, both HSV and CMV reactivation were associated with a longer duration of MV as compared to non-reactivation patients [29 (19.5–41) and 28 (20.5–37), respectively, vs. 17.5 (9–28) days; *p* < 0.05]. Co-reactivation patients had a longer duration of MV [58.5 (38–72.3); *p* < 0.05] and ICU stay [51.5 (32.5–69) vs. 27.5 (17.75–35.5) and 29 (20–30.5), respectively] as compared to patients with HSV or CMV reactivation alone. In multivariate analysis, HSV reactivation remained independently associated with a longer duration of MV and hospital length of stay.

**Conclusions:**

*Herpesviridae* reactivation is frequent among patients with severe ARDS under veno-venous ECMO and is associated with a longer duration of mechanical ventilation. The direct causative link between HSV and CMV reactivation and respiratory function worsening under ECMO remains to be confirmed.

## Background

Herpes simplex virus (HSV) and Cytomegalovirus (CMV) belong to the *Herpesviridae* family and are characterized by an often asymptomatic primo-infection generally during childhood followed by a latency phase. In immunocompromised subjects, *Herpesviridae* are common viral causes of opportunistic infections. But HSV and CMV reactivations are also frequently reported in intensive care unit (ICU) non-immunocompromised patients [1, 2]. Reactivation ranges from 13 to 64% and 15 to 45% for HSV and CMV, respectively [3, 4]. *Herpesviridae* reactivation in immunocompetent ICU patients is associated with poorer outcome [5]. HSV pulmonary reactivation has been described to be associated with a longer mechanical ventilation (MV) duration, ICU stay and mortality [2, 6, 7]. CMV reactivation is also associated with a higher mortality, MV duration and ICU length of stay [8]. In particular, CMV has been identified as a cause of persistent acute respiratory distress syndrome (ARDS) [9] and has also been shown to increase the mortality in ARDS patients [10]. However, despite these associations, the debate on the proper pathogen role of *Herpesviridae* rather than being a witness of patients’ severity is still ongoing. Studies failed to demonstrate that CMV prophylaxis was able to decrease Il-6 plasma levels in CMV seropositive critically ill patients [11] or to decrease mortality [12]. The role of *Herpesviridae* pre-emptive treatment among ICU patients has been recently evaluated in a randomized controlled trial (RCT) (NCT 02152358). The data concerning HSV showed that preemptive acyclovir did not decrease the duration of MV although a trend towards lower mortality was found in treated patients [13].

The most frequent risk factors for CMV and HSV reactivation in the ICU are patients severity, sepsis, prolonged MV [14], high-dose corticosteroid therapy, acute renal failure or massive transfusion [15], with a strong association for MV and sepsis [16]. Patients under veno-venous extracorporeal membrane oxygenation (VV ECMO) for severe ARDS [17] often combine several or all of these risk factors [18]. Despite the uncertainties regarding the exact role of *Herpesviridae* reactivation in immunocompetent critically ill patients, it might add to the pulmonary pathology in patients with ARDS. In experimental studies, CMV reactivation led to increased pulmonary fibrosis [19] and accessing bacterial pneumonia [20]. These findings suggest that *Herpesviridae*-related pulmonary pathology may be causally linked to the clinical disease course following ARDS onset, especially in the most severely ill patients who require prolonged mechanical ventilation, and might particularly concern patients under ECMO. However, despite the tight link that seems to exist between *Herpesviridae*, mechanical ventilation and ARDS, no study has investigated the occurrence of HSV and/or CMV reactivation in patients under VV ECMO. In this study, we aimed to assess the frequency of herpesviruses reactivation during ECMO course and to determine its impact on patients’ prognosis.

## Methods

### Study population

We conducted an observational, retrospective study in a medical ICU (ARDS and ECMO referee center) at the Marseille University Hospital between December 2011 and April 2017. Patients aged 18 or more, hospitalized in the ICU for severe ARDS requiring a VV ECMO for 2 days or more were included. HSV and/or CMV reactivation (see definition below) occurring after ECMO insertion was screened for these patients. Patients with immunosuppression (immunosuppressive treatments including corticosteroids > 0.5 mg/kg/day prednisone-equivalent within 30 days prior to inclusion, severe neutropenia < 0.5 G/L of neutrophils, HIV seropositivity, bone marrow or solid organ transplantation), antiviral therapy against HSV and/or CMV prior to inclusion, or HSV/CMV reactivation known at the time of ECMO insertion were excluded.

### HSV and CMV reactivation

At the time of the study, HSV and CMV screening were routinely performed twice weekly in all patients under MV.

HSV reactivation was defined by a positive qualitative throat sample (Virocult^®^) PCR.

CMV reactivation was defined by a positive quantitative blood PCR with a copy number > 500/ml.

When a broncho-alveolar lavage (BAL) was performed for suspicion of ventilator-associated pneumonia, HSV and CMV PCR were systematically realized in BAL and blood.

CMV viral loads were converted in IU/ml and qualified as “high reactivation” for viral loads greater than or equal to 1000 IU/ml or “low reactivation” for viral loads of 100–999 IU/ml [10].

CMV antigenemia was also researched in case of reactivation suspicion.

### Baseline assessment and data collection

The following data were retrospectively recorded from the patients’ medical file: age, sex, Simplified Acute Physiologic Score II (SAPS II) [21], Sequential Organ Failure Assessment (SOFA) score [22], presence of co-morbidities, presence of previous immunosuppression, cause of ARDS, date of MV initiation, date of ECMO implementation, other organ failure associated with ARDS during ICU stay (in particular need for catecholamines or renal replacement therapy), blood transfusion, post-aggressive pulmonary fibrosis (defined by an alveolar procollagen III higher than 9 µg/l) [23], time of HSV/CMV reactivation, delay between MV and HSV/CMV reactivation, delay between ECMO and HSV/CMV reactivation, duration of MV (from the day of intubation to the day of MV weaning), ECMO duration (from the day of ECMO implementation to its removal or death), ECMO-free days at day 28, ventilator-free days (VFD) at day 28, ICU length of stay [from the day of ICU admission (in the first ICU if the patient was referred from another hospital) to discharge], hospital length of stay [from the admission to hospital (in the original hospital if the patient was referred from another hospital) to discharge to home or to rehabilitation ward], ICU and hospital mortality, acyclovir or ganciclovir treatment after reactivation under ECMO.

### Statistical analysis

Statistical analysis was performed using IBM SPSS Statistics version 20.0 (IBM SPSS Inc., Chicago, IL, USA). First, a univariate analysis was performed. Data were expressed as mean ± the standard deviation or median with interquartile range for the quantitative variables, and as numbers and percentages for the categorical variables. Patient characteristics and clinical outcomes were compared to the viral reactivation status of the patients or antiviral treatment.

Groups were compared using the Chi-square or Fisher’s exact test for categorical characteristics, and using the Student’s *t* test or Mann–Whitney *U* test for continuous ones, as appropriate.

Then a multivariate analysis was performed to assess the independent effect of viral reactivation on different outcomes.

Multiple linear regression was used to construct models. Variables that were marginally significant (*p* < 0.10) in the univariate analysis, and that had clinical relevance were included in the regression models. Beta coefficients and their *p* values were presented.

A two-sided *p* value less than 0.05 was considered statistically significant.

## Results

**Fig. 1:**
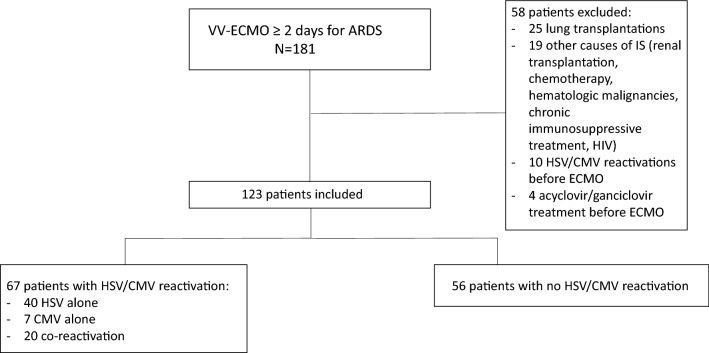
Study flowchart. *CMV* Cytomegalovirus, *HSV* herpes simplex virus, *IS* immunosuppression, *VV-ECMO* veno-venous extracorporeal membrane oxygenation

During the study period, 181 patients were admitted to our ICU for severe ARDS requiring a VV ECMO for 2 days or more (see flowchart, Fig. 1). Of these, 58 patients were excluded because of immunosuppression (44 patients), HSV/CMV reactivation at the time of ECMO implementation (10 patients) or acyclovir/ganciclovir treatment before ECMO (4 patients). Among the 123 patients included, 67 patients (54%) experienced HSV and/or CMV reactivation during the ICU stay and 56 (46%) were free from HSV/CMV reactivation at the time of ICU discharge or death.

### Patients’ characteristics

Population’s characteristics are presented in Table 1.

**Table 1:** Patients’ characteristics at ICU admission

	Reactivation (*n* = 67)	Non-reactivation (*n* = 56)	*p* value
Patient characteristics			
Age (years, mean ± SD)	52.9 (± 15.7)	50.6 (± 12.9)	0.39
Male gender (%)	49 (73)	38 (68)	0.66
Cancer (%)	10 (15)	5 (9)	0.46
Diabetes mellitus (%)	8 (12)	4 (7)	0.56
Congestive heart failure (%)	10 (15)	3 (5)	0.15
COPD (%)	15 (22)	7 (13)	0.24
Chronic renal failure (%)	3 (5)	0 (0)	0.25
Smoker (%)	42 (63)	26 (46)	0.10
Surgical reason for admission (%)	12 (18)	7 (13)	0.56
ARDS of primary pulmonary origin (%)	60 (90)	47 (84)	0.51
MV before ECMO (days) (median, 1st–3rd quartile)	4 (1–8)	1 (1–5)	< 0.01
Markers of disease severity at ICU admission			
IGS2 score (mean ± SD)	51.3 (± 15.2)	50.8 (± 16.5)	0.86
SOFA score (mean ± SD)	9.7 (± 4.0)	10.7 (± 4.1)	0.18
Septic shock (%)	58 (87)	46 (82)	0.67
Acute renal failure (%)	45 (67)	40 (71)	0.75

Data are presented as median and interquartile range or mean ± standard deviation or absolute value and percentage

Patients with HSV/CMV reactivation had a longer MV before ECMO than non-reactivated patients (*p *< 0.01).

### HSV/CMV reactivation

Among the 67 patients experiencing HSV/CMV reactivation under ECMO, 40 had HSV reactivation alone, 7 had CMV reactivation alone and 20 patients had both viruses’ reactivation (co-reactivation). HSV reactivation was diagnosed by throat sample in 38 (63%) patients and 21 (35%) patients had a positive PCR in BAL. Only 3 patients (5%) exhibited a positive HSV viremia. CMV reactivation was diagnosed in blood for 22 (81%) patients and 5 (19%) patients had a positive PCR in BAL. HSV reactivation occurred earlier after the beginning of MV than CMV [10 (6–15) vs. 19 (13–29) days; *p* < 0.01] and after ECMO implementation [4 (2–8) vs. 14 (10–20) days; *p* < 0.01].

Mean CMV viral loads (in blood or BAL) were 6916 ± 8934 IU/ml with a high reactivation for 25 (93%) patients.

### Clinical outcomes in reactivated and non-reactivated patients

Clinical outcomes are presented in Table 2.

**Table 2:** Clinical outcomes according to reactivation status

	Reactivation (*n* = 67)	Non-reactivation (*n* = 56)	*p* value
Duration of mechanical ventilation^a^ (days)	34 (22–52.5)	17.5 (9–28)	< 0.01
Ventilator-free days at D28^a^ (days)	0 (0–0)	0 (0–0)	0.16
Weaned from ECMO (%)	40 (60)	27 (48)	0.28
Duration of ECMO^a^ (days)	15 (10–22.5)	9 (5–14)	< 0.01
ECMO-free days at D28^a^ (days)	0 (0–16)	0 (0–17)	0.75
ICU mortality (%)	34 (51)	33 (59)	0.47
Hospital mortality (%)	35 (52)	33 (59)	0.58
ICU length of stay^a^ (days)	29 (19.5–47.5)	16 (9–30)	< 0.01
Hospital length of stay^a^ (days)	44 (29–63.5)	24 (11–43)	< 0.01
Pulmonary fibrosis (%)	16 (24)	8 (14)	0.27
Transfusion of blood products^a^	11 (7–21.5)	10 (6–15)	0.05

Data are presented as median and interquartile range or absolute value and percentage

^a^Median, 1st–3rd quartile

Patients exhibiting HSV/CMV reactivation received more transfusion [11 (7–21.5) vs. 10 (6–15) red cells pellets; *p* = 0.05]. Pulmonary fibrosis, diagnosed by an alveolar procollagen III > 9 µG/l, was not different between both groups.

In univariate analysis, HSV/CMV reactivation was associated with a longer duration of mechanical ventilation [34 (22–52.5) vs. 17.5 (9–28) days; *p* < 0.01], a longer duration of ECMO [15 (10–22.5) vs. 9 (5–14) days; *p* < 0.01], and a prolonged ICU [29 (19.5–47.5) vs. 16 (9–30) days; *p* < 0.01] and hospital stay [44 (29–63.5) vs. 24 (11–43) days; *p* < 0.01]. However, in multivariate analysis (Table 3), viral reactivation remained associated with prolonged MV only.

**Table 3:** Association between HSV/CMV reactivation and clinical outcomes

	Coefficient	*p* value
Mechanical ventilation duration		
Viral reactivation	4.01	< 0.01
ECMO duration		
Viral reactivation	1.64	0.32
ICU length of stay		
Viral reactivation	− 3.63	0.03
Hospital length of stay		
Viral reactivation	0.82	0.73

Multivariate analysis evaluating, after adjustment on patients’ severity and length of MV and ECMO duration before reactivation, the clinical impact of HSV/CMV reactivation. The coefficient designates the number of days by which the different endpoints are affected

When analyzing separately patients discharged alive from the ICU, duration of MV remained longer in the reactivation group [38 (28–54) vs. 27 (18–35) days; *p* < 0.01].

### Impact of HSV, CMV, and co-reactivation on clinical outcomes

When separating patients according to HSV, CMV, and co-reactivation (HSV and CMV), we found that HSV reactivation was associated with a longer duration of MV [29 (19.5–41) vs. 17.5 (9–28) days; *p* < 0.05] and ECMO [14 (9.75–20) vs. 9 (5–14) days; *p* < 0.05] as compared with non-reactivation patients. Patients with CMV alone also had a longer duration of MV as compared with non-reactivation patients [28 (20.5–37) vs. 17.5 (9–28) days; *p* < 0.05]. Co-reactivation patients had a longer duration of MV [58.5 (38–72.3) vs. 29 (19.5–41), 28 (20.5–37) and 17.5 (9–28) days, respectively; *p* < 0.05] and ICU stay [51.5 (32.5–69) vs. 27.5 (17.75–35.5), 29 (20–30.5) and 16 (8.75–30.25) days, respectively, *p* < 0.05] as compared to HSV, CMV, and non-reactivation patients. Co-reactivation patients had a longer ECMO duration as compared with non-reactivation patients [19.5 (10.75–33) vs. 9 (5–14) days; *p* < 0.05], a longer hospital length of stay as compared with HSV reactivation and non-reactivation patients [70 (43.5–87) vs. 35 (26.75–56) and 24 (11–43) days; *p* < 0.05] (Table 4).

**Table 4:** Clinical outcomes in HSV, CMV, HSV and CMV (co-reactivation) or non-reactivation groups

	HSV reactivation (*n* = 40)	CMV reactivation (*n* = 7)	HSV and CMV reactivation (*n* = 20)	Non reactivation (*n* = 56)
Duration of mechanical ventilation^e^ (days)	29 (19.5–41)^a,b^	28 (20.5–37)^a,b^	58.5 (38–72.3)^a,c,d^	17.5 (9–28)^b,c,d^
Ventilator-free days at D28^e^ (days)	0 (0–0)	0 (0–0)	0 (0–0)	0 (0–0)
Weaned from ECMO (%)	23 (58)	2 (29)	15 (75)	27 (48)
Duration of ECMO^e^ (days)	14 (9.75–20)^a^	13 (9.5–16)	19.5 (10.75–33)^a^	9 (5–14)^b,c^
ECMO-free days at D28^e^ (days)	0 (0–16)	0 (0–7)	5.5 (0–17.25)	0 (0–17)
ICU mortality (%)	20 (50)	5 (71)	9 (45)	33 (59)
Hospital mortality (%)	20 (50)	5 (71)	10 (50)	33 (59)
ICU length of stay^e^ (days)	27.5 (17.75–35.5)^b^	29 (20–30.5)^b^	51.5 (32.5–69)^a,c,d^	16 (8.75–30.25)^b^
Hospital length of stay^e^ (days)	35 (26.75–56)^b^	29 (23–44)	70 (43.5–87)^a,c^	24 (11–43)^b^

Data are presented as median and interquartile range or absolute value and percentage

^a^*p* < 0.05 compared with non-reactivation group

^b^*p* < 0.05 compared with HSV and CMV reactivation

^c^*p* < 0.05 compared with HSV reactivation

^d^*p* < 0.05 compared with CMV reactivation

^e^Median, 1st–3rd quartile

In multivariate analysis (Table 5), only HSV reactivation remained independently associated with a longer duration of MV and hospital length of stay but a shorter ICU stay.

**Table 5:** Association between HSV, CMV or HSV and CMV reactivation and clinical outcomes

	Mechanical ventilation duration	
	Coefficient	*p* value
HSV reactivation	4.23	< 0.01
CMV reactivation	2.89	0.32
HSV and CMV reactivation	3.54	0.11
ECMO duration		
HSV reactivation	2.14	0.26
CMV reactivation	2.23	0.51
HSV and CMV reactivation	3.88	0.14
ICU length of stay		
HSV reactivation	− 4	0.02
CMV reactivation	− 3.8	0.24
HSV and CMV reactivation	− 2.01	0.42
Hospital length of stay		
HSV reactivation	9.39	0.02
CMV reactivation	− 6.25	0.57
HSV and CMV reactivation	4.49	0.52

Multivariate analysis evaluating the clinical impact of HSV, CMV or HSV and CMV reactivation on MV and ECMO duration, ICU and hospital length of stay. The coefficient designates the number of days by which the different endpoints are affected

### Antiviral treatments

Thirty-four patients (51%) received an antiviral treatment (acyclovir or ganciclovir) during ECMO course. No difference in clinical outcomes was found between treated and untreated patients except a trend towards longer duration of MV for treated patients (Additional file 1: Table S1).

## Discussion

Until today, no data have been published concerning *Herpesviridae* reactivation in ICU patients under VV ECMO for severe ARDS. In this retrospective study covering a 5-year period, we found that HSV/CMV reactivation was frequent and concerned more than half non-immunocompromised patients, which is higher than that described in previous studies including all ICU patients [4, 8, 14]. This might be explained by several reasons: the use of PCR to diagnose reactivation with a higher sensitivity than older technics, the age of our cohort of patients (with a high probability of seropositivity for HSV and CMV at ICU admission) and the frequency of sepsis with a probable induced “immunoparalysis” [24]. In our cohort, HSV reactivation occurred earlier than CMV reactivation and the median time of reactivation for both viruses was comparable to what is described in “non-ECMO” patients [25]. CMV viral loads in blood and BAL were high in almost all patients. Elevated CMV viremia is associated with a higher risk of death or prolonged hospitalization [4].

Patients included were comparable except for the duration of MV before ECMO that was longer in the reactivation group. It is well known that MV is a risk factor for *Herpesviridae* reactivation with a strong association for CMV [16].

We found that *Herpesviridae* reactivation was associated with a prolonged MV, this association persisting in multi-variate analysis. We also found in these patients a prolonged ECMO duration, ICU, and hospital stay, although not confirmed in multivariate analysis. In a recently published meta-analysis, Li et al. [8] showed that CMV reactivation was associated with an increase of 9 days in MV and a 12 days increase in ICU stay. These results confirmed those published by Limaye et al. [4], which showed that CMV viremia among ICU patients was associated with a higher risk of death or prolonged ICU stay > 30 days. Similarly, in a case–control study [15], CMV reactivation was associated with a prolonged duration of MV and ICU stay. In a specific population of ARDS patients, Ong et al. [10] demonstrated that patients with CMV reactivation had a 15 (10–26) days median duration of MV as compared to 8 (6–12) days for non-reactivated patients. ICU length of stay was also longer [16 (11–28) vs. 9 (7–14) days] for reactivated patients. Same results have been published concerning HSV reactivation [2, 6], especially during ARDS. Our findings suggest that *Herpesviridae* reactivation is associated with worse outcomes for ARDS patients including when they are under ECMO. When examining the impact of each virus separately, we found that both HSV and CMV were associated with a prolonged MV, and also ECMO duration for HSV. Co-reactivation had a negative effect not only on MV and ECMO duration but also on ICU and hospital stay, as compared to patients free from reactivation or with only one virus. HSV was independently associated with a longer duration of MV and hospital length of stay but, surprisingly, a shorter ICU stay. These results highlight the potential negative role of HSV in ARDS patients under ECMO. Very recently, Luyt et al. [13] showed that preemptive treatment with acyclovir, compared to placebo, for mechanically ventilated patients with oropharyngeal HSV reactivation, was not associated with shorter MV duration. However, a trend towards lower day-60 mortality was observed in the acyclovir group. In our cohort, more than half of the patients were treated after the diagnosis of viral reactivation. Treatment with acyclovir or ganciclovir did not improve the outcomes, with a trend for longer duration of MV in the sub-group of treated patients. These results might be explained by the fact that anti-viral treatment was decided by clinicians more frequently in case of worsening respiratory status, persisting fever or end-organ HSV/CMV disease, and so reserved for the most severe patients. We did not find any increase in renal failure in patients receiving antiviral drugs, which was also noticed in Luyt et al.’s study [13]. However, we cannot exclude any other side effects.

Our study has some limitations. First, the retrospective design of our cohort, counterbalanced by the important number of patients included during this 5-year period. Second, the applicability of our results to the general population of patients under ECMO must be considered cautiously considering the high rate of patients treated with antiviral drugs after reactivation. However, in non-EMCO patients, routine screening of *Herpesviridae* has been reported as well as the use of antiviral treatment despite the lack of recommendation [3, 15, 16]. Third, few patients developed an isolated CMV reactivation. This precludes to conclude clearly on the specific impact of CMV in our cohort of patients. Fourth, our methods do not prevent competing risks. In particular, the difference in MV duration between reactivated and non-reactivated patients might have been influenced by the high mortality reported. However, this mortality was similar in both groups and the difference of MV duration persisted when considering only the patients discharged alive from the ICU.

Finally, despite the statistical association, it is not possible to conclude whether *Herpesviridae* reactivation is directly responsible for worse clinical outcomes or if it is a consequence and a witness of the severity of the disease, as in non-ECMO populations [3].

## Conclusions

*Herpesviridae* reactivation is frequent among patients with severe ARDS under veno-venous ECMO and is associated with a prolonged mechanical ventilation. This association is present for HSV as well as CMV and also for co-reactivation. The direct causative link between HSV and CMV reactivation and respiratory function worsening under ECMO remains to be confirmed.

## Supplementary information

**Additional file 1: Table S1.** Clinical outcomes according to anti-viral treatment.

## Data Availability

The datasets used and/or analyzed during the current study are available from the corresponding author on reasonable request.

## Abbreviations

ARDS: acute respiratory distress syndrome
BAL: broncho-alveolar lavage
CMV: Cytomegalovirus
ECMO: extracorporeal membrane oxygenation
HIV: human immunodeficiency virus
HSV: herpes simplex virus
ICU: intensive care unit
Il-6: interleukine 6
IU/ml: international units/milliliter
MV: mechanical ventilation
PCR: polymerase chain reaction
SAPS II: Simplified Acute Physiologic Score II
SOFA: Sequential Organ Failure Assessment
VFD: ventilator free days
Vs.: versus
VV: veno-venous
