# Supplementary material for: Herpes simplex virus and Cytomegalovirus reactivation among severe ARDS patients under veno-venous ECMO
Source: Ann Intensive Care. 2019 Dec 23;9:142. doi: 10.1186/s13613-019-0616-6 (PMC6928167; doi:10.1186/s13613-019-0616-6)
Supplement: Supplementary file 1 — Additional file 1: Table S1. Clinical outcomes according to anti-viral treatment. [file 13613_2019_616_MOESM1_ESM.docx]

**Table S1. Clinical outcomes according to anti-viral treatment**

|  | Anti-viral treatment  (n=34) | No anti-viral treatment (n=33) | *p* *value* |
| --- | --- | --- | --- |
| Duration of mechanical ventilation ^1^ (days) | 39 (27 – 62) | 29 (20 – 42) | 0.055 |
| Ventilator-free days at D28 ^1^ (days) | 0 (0 – 1) | 0 (0 – 8) | 0.12 |
| Weaned from ECMO (%) | 19 (56 ) | 21 (64) | 0.52 |
| Duration of ECMO ^1^ (days) | 18 (12 – 30) | 12 (10 – 19) | 0.07 |
| ECMO-free days at D28 ^1^ (days) | 0 0 – 12) | 12 (0 – 18) | 0.14 |
| ICU mortality (%) | 20 (59) | 14 (42) | 0.11 |
| Hospital mortality (%) | 21 (62) | 14 (42) | 0.1 |
| ICU length of stay ^1^ (days) | 33 (23 – 52) | 28 (18 – 37) | 0.1 |
| Hospital length of stay ^1^ (days) | 43 (29 – 79) | 44 (30 – 58) | 0.36 |

^1 :^ median, 1^st^ – 3^rd^ Quartile

Data are presented as median and interquartile range or absolute value and percentage.
